# Behavioral economic implementation strategies to improve serious illness communication between clinicians and high-risk patients with cancer: protocol for a cluster randomized pragmatic trial

**DOI:** 10.1186/s13012-021-01156-6

**Published:** 2021-09-25

**Authors:** Samuel U. Takvorian, Justin Bekelman, Rinad S. Beidas, Robert Schnoll, Alicia B. W. Clifton, Tasnim Salam, Peter Gabriel, E. Paul Wileyto, Callie A. Scott, David A. Asch, Alison M. Buttenheim, Katharine A. Rendle, Krisda Chaiyachati, Rachel C. Shelton, Sue Ware, Corey Chivers, Lynn M. Schuchter, Pallavi Kumar, Lawrence N. Shulman, Nina O’Connor, Adina Lieberman, Kelly Zentgraf, Ravi B. Parikh

**Affiliations:** 1 Perelman School of Medicine, University of Pennsylvania, 3400 Civic Center Blvd, 10S-113, Philadelphia, PA 19104 USA; 2 Penn Center for Cancer Care Innovation, Abramson Cancer Center, Penn Medicine, Philadelphia, PA USA; 3 Penn Implementation Science Center, Leonard Davis Institute of Health Economics, Philadelphia, PA USA; 4 Penn Medicine Nudge Unit, Center for Healthcare Innovation, Penn Medicine, Philadelphia, PA USA; 5 Center for Interdisciplinary Research on Nicotine Addiction, University of Pennsylvania, Philadelphia, PA USA; 6 School of Nursing, University of Pennsylvania, Philadelphia, PA USA; 7 Mailman School of Public Health, Columbia University, New York, NY USA

**Keywords:** Serious illness conversation, Advanced care planning, End-of-life cancer care, Pragmatic trials

## Abstract

**Background:**

Serious illness conversations (SICs) are an evidence-based approach to eliciting patients’ values, goals, and care preferences that improve patient outcomes. However, most patients with cancer die without a documented SIC. Clinician-directed implementation strategies informed by behavioral economics (“nudges”) that identify high-risk patients have shown promise in increasing SIC documentation among clinicians. It is unknown whether patient-directed nudges that normalize and prime patients towards SIC completion—either alone or in combination with clinician nudges that additionally compare performance relative to peers—may improve on this approach. Our objective is to test the effect of clinician- and patient-directed nudges as implementation strategies for increasing SIC completion among patients with cancer.

**Methods:**

We will conduct a 2 × 2 factorial, cluster randomized pragmatic trial to test the effect of nudges to clinicians, patients, or both, compared to usual care, on SIC completion. Participants will include 166 medical and gynecologic oncology clinicians practicing at ten sites within a large academic health system and their approximately 5500 patients at high risk of predicted 6-month mortality based on a validated machine-learning prognostic algorithm. Data will be obtained via the electronic medical record, clinician survey, and semi-structured interviews with clinicians and patients. The primary outcome will be time to SIC documentation among high-risk patients. Secondary outcomes will include time to SIC documentation among all patients (assessing spillover effects), palliative care referral among high-risk patients, and aggressive end-of-life care utilization (composite of chemotherapy within 14 days before death, hospitalization within 30 days before death, or admission to hospice within 3 days before death) among high-risk decedents. We will assess moderators of the effect of implementation strategies and conduct semi-structured interviews with a subset of clinicians and patients to assess contextual factors that shape the effectiveness of nudges with an eye towards health equity.

**Discussion:**

This will be the first pragmatic trial to evaluate clinician- and patient-directed nudges to promote SIC completion for patients with cancer. We expect the study to yield insights into the effectiveness of clinician and patient nudges as implementation strategies to improve SIC rates, and to uncover multilevel contextual factors that drive response to these strategies.

**Trial registration:**

ClinicalTrials.gov, NCT04867850. Registered on April 30, 2021.

**Funding:**

National Cancer Institute P50CA244690

**Supplementary Information:**

The online version contains supplementary material available at 10.1186/s13012-021-01156-6.

Contributions to the literature

- The majority of patients with cancer die without a documented serious illness conversation (SIC), despite evidence that SICs improve patient outcomes.
- This study will test the effect of implementation strategies informed by behavioral economics, involving the use of nudges to promote SICs.
- This study leverages rapid cycle approaches, pragmatic trial design, and mixed methods to evaluate the effect of nudges to clinicians, patients, or both, compared to usual care, on SIC completion, and to enhance understanding of factors that influence response to these strategies and their mechanistic underpinnings.

## Background

Patients with cancer often experience physical and emotional distress, utilize unplanned acute care, and undergo medical interventions that are discordant with their wishes at the end of life [1–6]. Serious illness conversations (SICs) that elicit patients’ values, goals, and care preferences, particularly earlier in the disease trajectory, are an evidence-based practice that improves patient mood and quality of life [7–12] and are recommended by national guidelines, including those of the American Society of Clinical Oncology and National Academies of Medicine [13–15]. However, most patients with cancer die without a documented SIC, contributing to unwarranted and unwanted aggressive care at the end of life [7]. Moreover, SICs are implemented inequitably. Those more likely to experience social and health inequities, such as Black or African American patients, are less likely than non-Hispanic White patients to have SICs [16–21]. Current implementation strategies [22] to promote SICs—including the Serious Illness Care Program (SICP) developed by Ariadne Labs [23]—focus primarily on clinician education and training and have only marginally increased the timeliness and frequency of SICs [11, 12]. While core elements of this program are scalable, such as its structured conversation guide for clinicians, SIC completion in routine practice remains low. For example, even after formal SICP training, medical oncology clinicians at our large academic cancer center documented SICs for fewer than 5% of patients with cancer seen in medical oncology practices [24].

Efforts to implement SICs can be enhanced using approaches from behavioral economics, a discipline that encompasses a set of principles, theories, and strategies derived from economics, cognitive psychology, and social psychology to understand human decision-making. Behavioral economics posits that individuals deploy common mental heuristics that may account for behavior that is “non-rational” (i.e., not consistent with maximizing individual utility). Clinician heuristics that may undermine the initiation of SICs include *optimism bias*, or the belief that one’s own patient is unlikely to experience a negative event; uncertainty about prognosis and optimal SIC timing; and fear that bringing up end-of-life issues may be distressing to patients [25, 26]. Due to *optimism bias*, clinicians routinely overestimate the life expectancy of patients with advanced cancer, particularly in the era of novel and personalized therapeutics [27–29], and delay SICs until too late in the disease course. Delaying SICs reinforces a *social norm* that SICs are not part of routine oncology care and are not appropriate until late in the disease course, often after unplanned acute care utilization [15, 30–32]. Such norms are powerful behavioral determinants: individuals desire to conform to an approved behavior (*an injunctive norm*) and the behavior of others (*a descriptive norm*). These norms may be influenced by broader factors such as structural racism—most notably, beliefs that non-White patients may be less receptive to end-of-life conversations—that contribute to inequitable access to SICs [33]. Scant research has evaluated strategies designed to harness norms directly and align clinicians and patients toward engaging in SICs [34].

Implementation strategies informed by behavioral economics are well-suited to address these barriers, which fundamentally imply the need for strategies directed towards clinicians and patients. By intentionally modifying the way choices are framed, *nudges* can counter behavioral pathways that might thwart behavior change or harness those pathways to redirect decisions toward better outcomes, while preserving principles of autonomy and choice [24, 35–37]. Nudges to clinicians have been shown to increase rates of evidence-based behaviors such as influenza vaccination [38–40]. We demonstrated the effectiveness of a clinician-directed nudge designed to counteract *optimism bias* and aid clinicians in identifying patients at high risk of predicted 180-day mortality based on a validated machine-learning prognostic algorithm (i.e., those most likely to benefit from SICs). This strategy led to a near fourfold increase in SIC documentation for high-risk patients, with particular improvement seen among non-White patients, and is now in routine use across sites at our large academic cancer center [25]. However, clinicians still did not have SICs for over half of high-risk patients, illustrating gaps that remain with a clinician-directed implementation strategy alone [25].

As a signature project of our National Cancer Institute-funded Penn Implementation Science Center in Cancer Control (P50 CA244690), this study will evaluate the effect of nudges to clinicians, nudges to patients, or nudges to both, on SIC completion. The clinician nudge will build on our prior approach to clinician nudges by adding performance feedback via peer comparison. This addition will serve to remind clinicians of their performance on SIC documentation by providing both an *injunctive norm* (institutional guidelines) and a *descriptive norm* (displaying the behavior of peers). The patient nudge will be a digital communication designed to normalize SICs as a routine part of cancer care and to prime patients (and, in turn, their clinicians) to have earlier SICs. *Priming* is a type of nudge that frames information to activate one’s self-efficacy and willingness to engage in behavior change [41] and may also signal an *injunctive norm* for both clinicians and patients. A prior study with patients that had serious illnesses (including some with cancer) demonstrated promise to this approach [42]. The clinician and patient nudges will be compared with usual care at our institution, which consists of a basic clinician-directed nudge identifying high-risk patients and providing performance feedback *without peer comparison*; importantly, this arm will serve as an active control. We hypothesize that providing a clinician nudge that counteracts optimism bias and targets injunctive and descriptive norms via peer comparison, and a patient nudge that normalizes and primes patients towards serious illness communication, will lead to increased rates of SICs for patients with cancer at risk of short-term mortality [35, 43, 44].

## Methods

### Study aims

The main objective of this four-arm cluster randomized pragmatic trial is to test the independent effects of multilevel behavioral economic implementation strategies involving nudges to clinicians, nudges to patients, or nudges to both, as compared to usual care, on SIC completion for patients predicted to be at high risk of 6-month mortality (aim 1). We hypothesize that each of the implementation strategy arms will increase SIC rates compared to usual care and that the combination arm (nudges to both clinicians and patients) will be most effective. We will also conduct a quantitative evaluation using secondary data to identify moderators of implementation effects on SIC completion, evaluating for heterogeneity of effects by patient race/ethnicity, income, and geographic location, as well as by clinician and practice factors (aim 2). Finally, we will investigate potential mechanisms by which the nudges increase SICs using qualitative data collected from clinicians and patients (aim 3).

### Study design

We will conduct a four-arm cluster randomized pragmatic trial to test the effect of nudges to clinicians, nudges to patients, or nudges to both, compared to usual care, on SIC completion. Using a 2 × 2 factorial design, eligible clinicians and patients will be randomized independently, yielding four study arms (Table 1). Clinician clusters will be randomized to receive a weekly nudge delivered via email and text message containing (a) identification of patients at high risk of predicted 6-month mortality and (b) performance feedback on SIC documentation compared to peers, versus usual care. Patients will be randomized to receive a nudge delivered via email and text message containing (a) a normalizing message and (b) a short questionnaire designed to prime patients to have earlier SICs, versus usual care. Usual care will consist of a basic clinician-directed nudge identifying high-risk patients and providing performance feedback *without peer comparison*, which will serve as an active control. The study arms are described in more detail below.

**Table 1:** Randomized 2 × 2 factorial design yielding 4 independent study arms

		Patient	
		*No nudge*	*Nudge*
**Clinician**	Nudge	**Arm 1: Nudge to clinician only** Identification of high-risk patients + performance feedback *with* clinician peer comparison	**Arm 3: Nudge to clinician and patient** Strategies from arms 1 and 2 in combination
	No nudge	**Arm 4: Usual care (active control)** Identification of high-risk patients + performance feedback without clinician peer comparison	**Arm 2: Nudge to patient only** Normalizing message + patient priming questionnaire

**Fig. 1:**
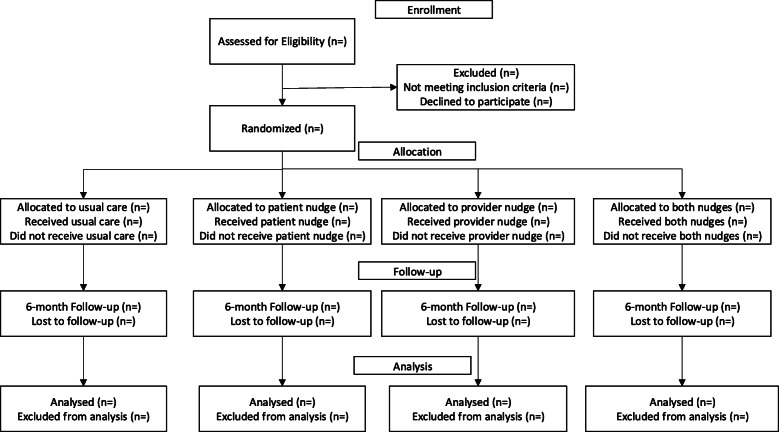
CONSORT diagram

The study duration for clinicians will be approximately 12 months, during which they will receive nudges according to the assigned study arm. The study duration for patients will be approximately 6 months per participant, defined by an initial index clinical encounter (marking study entry and the beginning of the nudge exposure period according to the assigned study arm) and by a 6-month follow-up period over which study outcomes will be ascertained (Fig. 1).

### Participants and setting

The study participants will include approximately 5500 high-risk patients with cancer cared for by approximately 166 medical and gynecologic oncology clinicians across the following four hospitals and six community practice sites within the Penn Medicine Abramson Cancer Center (ACC): Hospital of the University of Pennsylvania, Pennsylvania Hospital, Penn Presbyterian Medical Center, Chester County Hospital, Valley Forge Medical Center, Radnor Medical Center, Cherry Hill Medical Center, Voorhees Medical Center, Sewell Medical Center, and Regional Hematology Oncology Associates. These entities include approximately 200 oncologists and annually serve >15,000 new cancer patients per year (52% female, 42% Hispanic, 16.2% Black, 21% Medicaid/uninsured).

The clinician sample will include eligible medical oncologists, gynecologic oncologists, and advanced practice providers (APPs, i.e., physician assistants and nurse practitioners) caring for patients with cancer at participating sites. Eligible clinician participants will provide care at least 1 half-day clinic session per week for adult (age > 18 years) patients with solid, hematologic, or gynecologic malignancies at a participating site. Clinicians providing exclusively survivorship, genetics, benign hematology, leukemia, or bone marrow transplant care will be excluded from the clinician sample. We justify these exclusions by (1) the lack of high-risk patients in survivorship, genetics, and benign hematology clinics and (2) the suboptimal algorithm performance among patients with leukemia or after bone marrow transplantation. All eligible clinicians will have the opportunity to undergo SICP training, which is required training for medical and gynecology clinicians at our health system. Table 2 details study inclusion and exclusion criteria.

**Table 2:** Participant inclusion and exclusion criteria

**Clinicians**	
*Inclusion*	*Exclusion*
Medical oncologists, gynecologic oncologists, and advanced practice providers (APPs, i.e., physician assistants, nurse practitioners)	Provide exclusively survivorship, genetics, benign hematology, leukemia, or bone marrow transplant care
Provide care at least 1 clinic session per week for adult (age > 18 years) patients with solid, hematologic, or gynecologic malignancies at a participating Penn Medicine practice site	
**Patients**	
*Inclusion*	*Exclusion*
Receive care for a solid, hematologic, or gynecologic malignancy from an eligible clinician at a participating Penn Medicine practice site	Have a previously documented SIC within 6 months of enrollment
Have at least one scheduled outpatient clinical encounter (either in person or via telemedicine) during the study period	Have a non-valid mobile phone number

The patient sample will include those receiving care for solid, hematologic, or gynecologic malignancies from an eligible clinician at a participating site, who have at least one scheduled outpatient clinical encounter either in person or via telemedicine during the study period (“index clinical encounter”). We will exclude patients with a previously documented SIC within 6 months of enrollment or with a non-valid mobile phone number, representing approximately 5% of the cohort. Patients will accrue as they are seen in follow-up by an eligible clinician at a participating practice site.

This study was approved by the University of Pennsylvania Institutional Review Board and by the other sites under a reliance agreement. Since this is a pragmatic trial focused on improving implementation of evidence-based practices with minimal risk to patients, we received a waiver of participant informed consent for clinicians and patients for aims 1 and 2. For aim 3, potential participants will provide informed consent prior to data collection.

### Study procedures and implementation strategies

Eligible clinicians and patients will be identified using the criteria listed above. Eligible clinicians will be randomized in clusters at the start of the study and will receive nudges identifying high-risk patients with or without peer comparisons for the duration of the study period. Clinicians will be clustered with APPs with whom they work, and vice versa, and these non-overlapping clusters will serve as the clinician unit of randomization and analysis. Clusters of clinicians, once identified, will be randomized to study arms by small-block permutation.

Eligible patients will be randomized in advance of a qualifying index clinical encounter and will accrue to the study accordingly. Patients will be exposed to implementation strategies based on their assigned study arm, which will depend on their own randomization (determined at the time of the index clinical encounter) and that of their clinician (determined at study initiation). Randomization will therefore employ a hybrid model of cluster randomization of clinician groups and independent randomization of patients.

We employed rapid cycle approaches to finalize the content, messaging, and design to optimize and refine implementation strategies prior to trial launch. These rapid cycle iterations included design meetings with behavioral economics experts (co-authors DAA and AMB), in-depth discussion with oncology clinicians who subspecialize in a variety of cancer subtypes and who were recruited from ten practice sites, and focus group discussions with members of a patient and caregiver cancer advisory group. We then engaged in multiple rounds of usability testing with both clinicians and patients, resulting in the final patient and clinician nudges as described below.

#### Nudge to clinician only

**Fig. 2:**
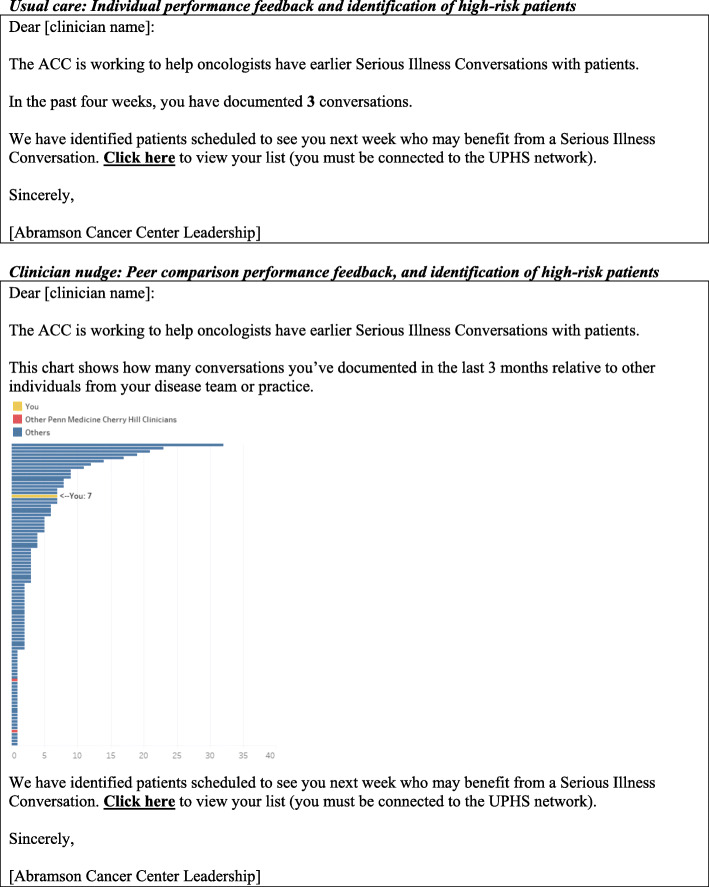
Clinician nudge

The clinician nudge will be delivered weekly via email with text message reminders sent on the mornings of clinic sessions. The weekly emails will identify patients scheduled for outpatient visits in the following week who are at high risk of predicted 180-day mortality based on a validated machine-learning prognostic algorithm [45, 46]. The emails will also contain clinician performance feedback on SIC documentation over the past 4 weeks with graphical comparison to peer clinicians who specialize in their specific disease group (e.g., thoracic, genitourinary, etc.) and/or who practice at their practice site. The text messages sent on the mornings of clinic sessions will alert clinicians to the appointment time and initials of high-risk patients scheduled to be seen during the clinic that day. Figure 2 shows the sample content of the clinician messaging.

#### Nudge to patient only

**Fig. 3:**
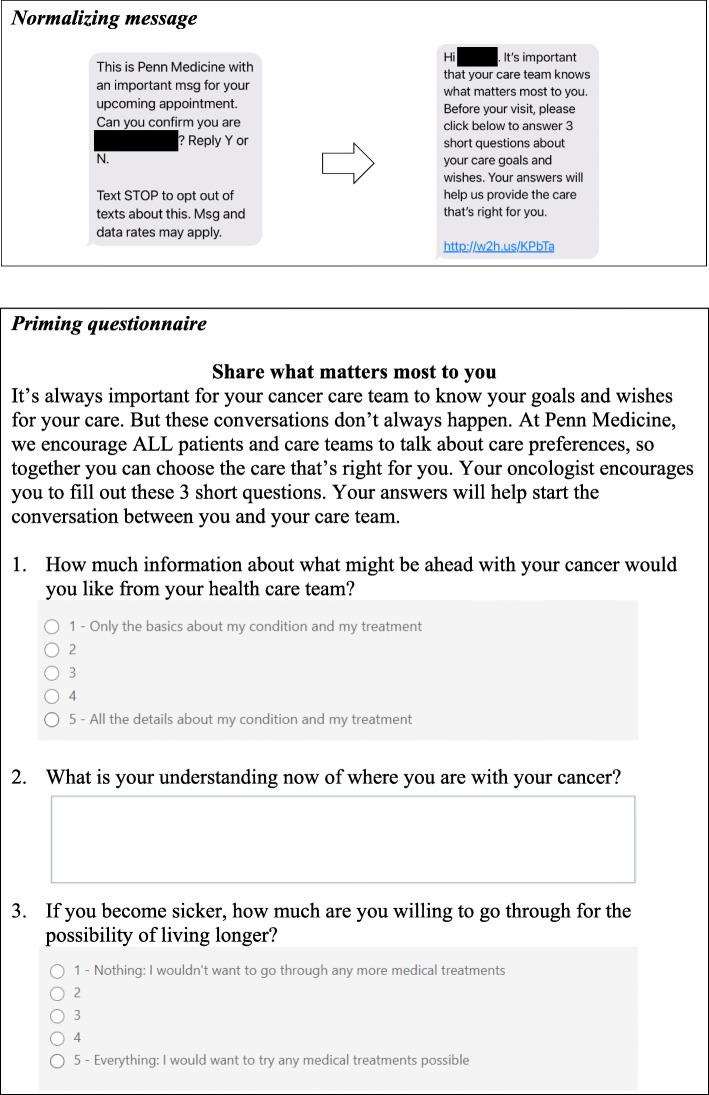
Patient nudge

The patient nudge will be delivered via text message and email in advance of the index clinical encounter. It will consist of a normalizing message prompting patients to complete a brief electronic questionnaire designed to prime patients towards having a SIC and will be re-sent a maximum of two times at monthly intervals only for those patients who neither fill out the priming questionnaire nor have a documented SIC during follow-up. Patients will have the opportunity to opt out of receiving any further text messages at any point. Patient-reported data from the priming questionnaire will be shared with the appropriate clinical team in real-time via the electronic medical record. Figure 3 shows the sample content of the patient messaging.

#### Nudges to both clinician and patient

Both strategies described above will be used in combination.

#### Usual care

Usual care at our institution changed as a result of our prior study, discussed above, showing the effectiveness of a clinician-directed nudge to promote SICs [25]. Accordingly, usual care in this trial will consist of weekly emails and text messages identifying high-risk patients, as well as clinician performance feedback *without peer comparison*. This approach has the advantage of incorporating an active control arm and will also enable us to understand the effect of clinician performance feedback with versus without peer comparison. Figure 2 shows the sample content of the clinician nudge used in usual care.

### Measures

The primary and secondary outcomes are summarized in Table 3. The primary outcome is SIC documentation among high-risk patients only (as identified by the machine-learning algorithm), measured as the presence or absence of a standardized SIC note in the electronic medical record during a participant’s 6-month follow-up period after the index clinical encounter. SIC documentation by clinicians will serve as a proxy measure for completed SICs, but is also an important outcome by itself as documentation facilitates centralized communication of patient goals and wishes to the health care team. The secondary outcomes are SIC documentation, among all patients (regardless of algorithm-predicted mortality risk); outpatient palliative care referral, among high-risk patients only; and aggressive end-of-life care (composite of any of the following three criteria: chemotherapy within 14 days before death, hospitalization within 30 days before death, or admission to hospice 3 days or less before death), among high-risk patients who die. These secondary outcomes will each be measured at the patient level as a binary outcome, ascertained over a participant’s 6-month follow-up period.

**Table 3:** Primary and secondary outcomes

***Primary outcome***	***Patient population***	***Description***	***Ascertainment***
SIC documentation	High-risk patients	Measured at patient level as binary outcome, defined by the presence of a documented note within the electronic medical record (EMR) using a standardized SIC template	6-month period following index clinical encounter
***Secondary outcomes***	***Patient population***	***Description***	***Ascertainment***
SIC documentation	All patients	Measured at patient level as binary outcome, defined as above	6-month period following index clinical encounter
Palliative care referral	High-risk patients	Measured at patient level as binary outcome, defined as encounter with palliative care clinician	
Aggressive end-of-life care	High-risk decedents	Measured at patient level as a binary outcome, defined as including any of: - Chemotherapy within 14 days before death - Hospitalization within 30 days before death - Admission to hospice 3 days or less before death	

We will additionally collect quantitative data on potential moderators of implementation effects and any health inequities that arise during implementation [47], using data collected routinely through the electronic medical record, publicly available data from the US Census, and data from the clinician survey. These will include *patient-level variables* (age, sex, race/ethnicity, cancer type, cancer stage, and health insurance), *clinician-level variables* (years in practice and patient panel size), *practice-level variables* (community vs. hospital-based setting, urban vs. non-urban location, and payer mix), and *neighborhood-level variables* (linked at the patient- and practice-level, including median income and educational attainment). The baseline clinician survey will assess distal constructs (e.g., organizational learning) and proximal constructs (e.g., perceived self-efficacy to engage in SICs), given findings that these constructs are important for implementation [48].

Finally, following active trial participation, semi-structured interviews will assess contextual factors across the five domains of the Consolidated Framework for Implementation Research (CFIR) to understand patient and clinician experiences with and responses to the nudges. Among clinicians, a core interview guide comprised of semi-structured questions will be used to assess multilevel organizational or system factors and processes related to implementation of SICs. Specific questions will probe clinicians about key barriers and facilitators of the nudges. Among patients, questions will probe participants about reactions to the nudge and experiences at the provider and systems levels (e.g., perceptions of acceptability, appropriateness); additionally, in line with our health equity focus [49, 50], there will be questions about social and structural factors that may contribute to health inequities such as experiences of racism, discrimination, medical mistrust, perceived health care access, and language barriers. In addition to semi-structured interview questions, all clinicians and patients interviewed will complete a brief questionnaire that assesses demographics and beliefs and behaviors related to SICs.

### Sample size and power

We are seeking to detect the main effects of nudges and their interaction with at least 80% power using a two-sided alpha of 0.05. We anticipate enrolling 66 physician-APP clusters who will together care for an estimated 5500 high-risk patients over the study period, yielding approximately 83 high-risk patients per cluster on average. We have generated power calculations using various patient enrollment and cluster correlation assumptions (Table 4). We calculated power by simulation, generating exponential time to event under a variety of assumptions. Patients are clustered within clinician units. Within-cluster correlation was imposed by drawing correlated, normally distributed random numbers, transformed to exponential using copula methods.

**Table 4:** Power simulations

Patients per cluster	Within-cluster correlation	Detectable true effect		
		Clinician nudge [HR (power)]	Patient nudge [HR (power)]	Interaction [HRR (power)]
70	0.1	1.6 (86%)	1.25 (84%)	1.6 (80%)
70	0.3	2.0 (85%)	1.3 (90%)	1.8 (91%)
90	0.1	1.6 (91%)	1.25 (86%)	1.6 (85%)
90	0.3	2.0 (86%)	1.25 (84%)	1.6 (83%)

Assuming conservatively that we enroll 70 patients per cluster with a within-cluster correlation of 0.1, we will have >80% power to detect a true hazard ratio (*HR*) of effect of 1.6 for the clinician nudge, *HR* 1.25 for the patient nudge, and hazard ratio of ratios (*HRR*) of 1.6 for the interaction. Assuming 70 patients per cluster and a within-cluster correlation of 0.3, we will have >80% power to detect a true hazard ratio of effect of *HR* 2.0 for the clinician nudge, *HR* 1.3 for the patient nudge, and *HRR* 1.8 for the interaction. These estimates improve slightly if we assume enrollment of 90 patients per cluster, allowing us to detect similar effect sizes with more power, as shown in Table 4.

### Statistical methods

For aim 1, the primary outcome will be modeled in a time-to-event analysis using a Cox proportional hazards model, with cluster-correlated robust standard errors to account for patient clustering within clinician clusters. SIC documentation, as defined above, will serve as the primary event outcome. Days between the index clinical encounter and SIC documentation will serve as the time variable. Nudge exposure will be determined based on randomization of the clinician cluster and randomization of the patient. Cox regression will estimate hazard ratios for the main effects and a ratio of hazard ratios for the interaction of dual clinician and patient nudges. Significance will be determined using the *z*-score corresponding to each of the estimated effects. All enrolled patients will be included in the intention-to-treat analysis, and subjects will be censored at the time of last structured electronic health record activity or death, should they not be observed to have a documented SIC during the follow-up period. Covariates will be assessed across study arms and included in the Cox model if unbalanced across arms.

We will use our fitted Cox model to generate predicted probabilities of SIC documentation within 6 months, via marginal standardization, as well as median time to documented SIC across arms. The functional form of the model will be checked using cumulative martingale residuals, and the proportional hazards assumption will be checked using scaled Schoenfeld residuals. The secondary outcomes will be similarly modeled. All hypothesis tests will use a two-sided alpha of 0.05 as the threshold for statistical significance.

For aim 2, we will explore the heterogeneity of implementation effects on SIC documentation by including an interaction term between study arm and, separately, patient, clinician, and neighborhood factors. Evidence for effect modification will be judged based on the *z*-score corresponding to the ratio of hazard ratios (interaction term).

For aim 3, we will use convergent mixed-methods design and analysis to help identify *for whom* implementation strategies are most effective, including among patients more likely to experience social and health inequities, and to identify *how* strategies might work (i.e., mechanism of change) [51, 52]. Informed by CFIR, we will identify contextual conditions (e.g., inner setting) and implementation conditions (characteristics of specific implementation strategy and process) shaping response to patient and clinician nudges. The constant comparative method, guided by grounded theory [53, 54], will be used to deductively code a priori domains of interest (guided and organized by CFIR domains) and to inductively explore emergent findings and overarching themes. We will triangulate these qualitative data with other quantitative data collected in the trial (e.g., trial outcomes, structured questionnaire data). These coded data will serve as inputs to assess multilevel mechanisms shaping nudge effectiveness across our trial using qualitative comparative analysis.

## Discussion

This randomized controlled trial will test the effect of nudges to clinicians, patients, or both, versus usual care, as implementation strategies to improve SIC completion among patients with cancer at high risk of mortality. By harnessing heuristics of *both* clinicians and patients and thereby addressing multilevel barriers to SIC engagement, it builds on our prior work demonstrating the moderate effectiveness of a clinician-directed nudge alone [25]. Through incorporation of this basic clinician nudge into an active control arm, this study is uniquely positioned to assess the added impact of clinician peer comparison within the clinician nudge, as well as the impact of a patient nudge designed to normalize SICs and prime patients (and clinicians) to participate in them.

We expect the study to yield essential insights into the effectiveness of patient-level nudges, clinician-level nudges, or both as implementation strategies to increase the uptake of evidence-based practices in cancer care, and to advance our understanding of the multilevel contextual factors and mechanisms that drive response to these strategies. Specifically, we will advance the science of implementation by exploring patient, clinician, and inner-setting factors that contribute to response to patient and/or clinician nudges, including assessing effectiveness across a diverse group of patients and practice settings. We expect that this will lend critical insights into the external validity of these novel approaches. Furthermore, if successful, this study will provide a generalizable approach to integrate predictive analytics with nudges to engage both clinicians and patients in evidence-based conversations about treatment goals and end-of-life wishes. These results will lay the foundation for how care settings can ensure that patients with cancer and their clinicians engage in more frequent and earlier SICs and feel empowered to initiate optimally timed conversations. This may help drive goal-concordant care, improve quality of life, and reduce unwanted end-of-life care utilization. Furthermore, our study will enhance understanding of the extent to which clinician and/or patient nudges affect health inequities in cancer care delivery.

Our study has several limitations. First, this is a single-health system study and thus our approach, even if successful, may not generalize to other health systems—particularly institutions without robust electronic medical record infrastructure, clinician training on serious illness communication, or buy-in from administrative leaders and the clinical workforce. Despite this limitation, our study consists of a racially and ethnically diverse patient population, spanning academic and community oncology settings with a common electronic medical record infrastructure, and thus lessons should generalize to diverse settings. Second, our primary outcome of SIC documentation is not a robust measure of SIC quality or patient satisfaction. While SIC documentation has been used as an outcome in several supportive care studies [12, 25], future studies of nudges should assess patient-reported outcomes and other conversation quality metrics as their primary outcome; we will measure such outcomes in an exploratory fashion as part of aim 3.

If successful, future directions of this work include a large, multi-health system study to test the generalizability of patient and/or clinician nudges to improve SIC rates and a well-powered study to assess the impact of clinician and patient SIC nudges on end-of-life care utilization and outcomes.

## Supplementary Information

**Additional file 1.** CONSORT Checklist-SIC Protocol. Title: CONSORT 2010 checklist of information to include when reporting a randomized trial. Description: CONSORT checklist

**Additional file 2.** Full Protocol-SIC Protocol. Title: Social and Behavioral Sciences Human Research Protocol. Description: IRB-approved study protocol

**Additional file 3.** Funding Letter-SIC Protocol. Title: Notice of Award. Description: NCI funding letter

**Additional file 4.** IRB Approval Letter-SIC Protocol. Title: IRB Amendment: Notice of Approval. Description: IRB approval letter

## Data Availability

The datasets generated and/or analyzed during the current study are not publicly available due to patient privacy but are available from the corresponding author on reasonable request.

## Abbreviations

ACC: Abramson Cancer Center of Penn Medicine
ACP: Advanced Care Planning
APP(s): Advanced practice provider(s)
*HR*: Hazard ratio
*HRR*: Hazard ratio of ratios
NCI: National Cancer Institute
SIC(s): Serious illness conversation(s)
