# Supplementary material for: Behavioral economic implementation strategies to improve serious illness communication between clinicians and high-risk patients with cancer: protocol for a cluster randomized pragmatic trial
Source: Implement Sci. 2021 Sep 25;16:90. doi: 10.1186/s13012-021-01156-6 (PMC8466719; doi:10.1186/s13012-021-01156-6)

**Social and Behavioral Sciences Human Research Protocol**

**PRINCIPAL INVESTIGATORS:**

Ravi Parikh, MD, MPP, FACP

Assistant Professor, Medical Ethics and Health Policy and Medicine

Perelman School of Medicine, University of Pennsylvania

3400 Civic Center Boulevard, Philadelphia PA 19104

[Ravi.Parikh@pennmedicine.upenn.edu](about:blank)

Samuel U. Takvorian, MD, MS

Instructor of Medicine, Division of Hematology & Oncology

Perelman School of Medicine, University of Pennsylvania

3400 Civic Center Boulevard, Philadelphia, PA 19104

[Samuel.Takvorian@pennmedicine.upenn.edu](about:blank)

**PROTOCOL TITLE:**

Effect of behavioral nudges to clinicians, patients, or both on Serious Illness Conversation documentation for patients with cancer

**IRB NUMBER:**

844816

**NIH GRANT NUMBER:**

P50 CA244690

**VERSION**

V1: P50 ISC3 SPP2 protocol 12.14.2020 FINAL

V2: Protocol_V2_06.10.2021_Clean_Final

V3: Protocol_V3_07.26.2021_Clean_Final

**INTRODUCTION AND PURPOSE:**

Patients with cancer often experience physical and emotional distress, utilize unplanned acute care, and undergo medical interventions that are discordant with their wishes.^1–6^ Given the COVID-19 pandemic, these adverse outcomes are amplified, particularly for racial/ethnic minorities. Serious illness conversations (SICs) that elicit patients’ values, goals, and care preferences, particularly early in the disease trajectory, are an evidence-based practice, improve patient mood and quality of life,^7–12^ and are recommended by national guidelines.^13–15^ Preliminary data suggests that SICs among patients with cancer are associated with improved quality of life, increased hospice utilization, and decreased acute care utilization. However, most patients with advanced cancer die without a documented SIC and there are well-documented health disparities in implementation for racial and ethnic minorities.^16–21^ Current strategies to promote SICs, including the multi-component strategies of the *Serious Illness Conversation Program*, focus primarily on clinician education and have marginally increased the timeliness and frequency of SICs, and reduced patient anxiety and depression.^11,12^ While core elements of this program are transferable—such as its structured guide—clinical use remains low. For example, even after training, clinicians at Penn Medicine document SICs for fewer than 5% of patients with advanced cancer.^22^ There is critical need to develop, test, and disseminate strategies to improve the frequency of SICs.

Implementation strategies informed by behavioral economics are ideally suited to address this problem, which is fundamentally one of clinician and patient behavior change. By intentionally modifying the way choices are framed, behavioral nudges can lead to desirable changes in clinician behavior while preserving clinician choice.^22–25^ Our preliminary work demonstrates the effectiveness of an implementation strategy focusing on a clinician nudge, consisting of performance feedback and targeted text messages identifying patients at high risk of mortality based on a validated machine learning prognostic algorithm.^26^ This strategy led to a threefold increase in SIC documentation for high-risk patients, equitably across racial/ethnic minority subgroups, and is now in routine use across Penn Medicine practice sites. However, clinicians still did not document SICs for over half of patients, illustrating the limitations of a clinician-directed implementation strategy alone.^26^ The purpose of this study is to test behavioral economics-informed multilevel implementation strategies to prompt clinicians and patients to engage in early SICs. This study will expand on these preliminary findings to evaluate the synergy between clinician- *and* patient-directed nudges to increase SIC documentation.

**OBJECTIVES:**

Aim 1: Conduct a pragmatic four-arm randomized controlled trial to test the effectiveness of nudges to clinicians, nudges to patients, or nudges to both in increasing the frequency and timeliness of SIC documentation, compared to usual practice.

1. ***Primary objective:***

Test the effect of nudges to clinicians, nudges to patients, or nudges to both, compared to usual practice, on SIC documentation, among high-risk patients.

1. ***Secondary objectives:***

Test the effect of nudges to clinicians, nudges to patients, or nudges to both, compared to usual practice, on the following secondary outcomes:

1. SIC documentation, among all patients
2. Palliative care referral, among high-risk patients
3. Aggressive end-of-life care (any of the following three criteria: chemotherapy within 14 days before death, hospitalization within 30 days before death, or admission to hospice 3 days or less before death), among high-risk patients who die

Aim 2: Conduct a quantitative evaluation using secondary data (obtained via EMR and census, for patients, and by survey, for clinicians) to identify moderators of implementation effects on SIC documentation. [Note: the clinician survey will be conducted within a separate study funded by this grant and has been submitted for IRB approval as a separate protocol; data across the studies will be linked by clinician name and study ID.]

1. ***Primary objective:***

Evaluate for heterogeneity of implementation effects on SIC documentation by race/ethnicity, income, and geographic location as well as by clinician and inner setting factors.

**BACKGROUND AND RATIONALE:**

Serious illness conversations (SICs) that elicit patients’ values, goals, and care preferences, particularly early in the disease trajectory, are an evidence-based practice, improve patient mood and quality of life,^7–12^ and are recommended by national guidelines.^13–15^ However, most patients with advanced cancer die without a documented SIC and there are well-documented health disparities in implementation for racial and ethnic minorities.^16–21^ Clinician barriers to initiating SICs include optimism bias, or the belief that one’s own patient is unlikely to experience a negative event; uncertainty about prognosis and appropriate timing; and fear that bringing up end-of-life issues may be distressing to patients.^26,27^ Patient barriers to SIC initiation include fear of discussing the end of life and beliefs that SICs are not appropriate until late in the course of cancer.^15^ While previous studies have tested financial incentives for SIC documentation, little research has evaluated behavioral economics-informed strategies to align *both* clinicians and patients towards earlier SICs.

*Rationale for clinician nudge using mortality prediction and peer comparison.* Due to optimism bias, clinicians routinely overestimate the life expectancy of patients with advanced cancer^28,29^ and delay SICs until too late in the disease course. In part because of this, clinicians reinforce a social norm that early SICs are not part of routine oncology care. Providing an objective assessment of predicted mortality risk may help counteract optimism bias among clinicians, and help them identify patients most likely to benefit from SICs. Moreover, that individuals desire to conform to an approved behavior (an injunctive norm) and the behavior of others (a descriptive norm) may contribute to low observed SIC rates, and may also afford an opportunity for intervention. We expect that periodically reminding clinicians of their own performance on SIC documentation, while providing both an injunctive norm (citing national and institutional guidelines) and a descriptive social norm (displaying the behavior of their best performing peers), will lead clinicians to conform more closely to these norms, as has been shown in studies conducted in other contexts.^23,30,31^

*Rationale for patient nudge using priming.* Priming is a type of nudge that frames information to activate one’s self-efficacy and willingness to engage in behavior change*.*^32^ This type of nudge has not previously been evaluated as a tool to promote SICs for patients with cancer. We will test the added impact of a patient nudge designed to prime patients and, in turn, their clinicians to having a SIC.

**CHARACTERISTICS OF THE STUDY POPULATION:**

1. ***Target Population and Accrual:***

The target population includes approximately 5,500 high-risk patients with cancer cared for by approximately 166 medical and gynecologic oncology clinicians at the following hospital and free-standing community practice sites of the Penn Medicine Abramson Cancer Center (ACC), referred to hereafter as the “Implementation Laboratory”: Hospital of the University of Pennsylvania (HUP), Pennsylvania Hospital (PAH), Penn Presbyterian Medical Center (PPMC), Chester County Hospital (CCH), Valley Forge Medical Center, Radnor Medical Center, Cherry Hill Medical Center, Voorhees Medical Center, Sewell Medical Center and Regional Hematology Oncology Associates. The trial will be conducted pragmatically, and patients will accrue as they are seen in follow-up at a participating practice site by an eligible provider.

1. ***Key Eligibility Criteria:***

*Key inclusion criteria*

Eligible clinicians (M.D., P.A., or N.P.) must meet the following criteria:

- Provide care at least 1 clinic session per week for adult (age >18 years) patients with solid, hematologic, or gynecologic malignancies at a participating PennMedicine Implementation Lab site

Eligible patients must meet the following criteria:

- Receive care for a solid, hematologic, or gynecologic malignancy from an eligible provider at a participating PennMedicine Implementation Lab site
- Have at least one scheduled Return Patient Visit (either in person or via telemedicine) with an eligible PennMedicine provider during the study period

*Key exclusion criteria*

Clinicians will be ineligible for ANY of the following reasons:

- Provide exclusively benign hematology, leukemia or bone marrow transplant, survivorship, or genetics care

Patients will be ineligible for ANY of the following reasons:

- Previously documented SIC within 6 months
- Have a non-valid phone number

***3. Subject Recruitment and Screening:***

Given the pragmatic nature of this study, assessing the impact of implementation strategies delivered to clinicians and patients through adjustments to existing clinical workflows using the electronic medical record and other existing patient communication tools, ***we are requesting a waiver of informed consent***. There are several reasons to justify this request. First, it is not feasible to consent every patient and clinician randomized to nudges. Second, if members of the control group were consented, they would know they were being studied and this could affect their behavior, thereby complicating the study design and interpretation of its findings. Third, none of the nudges being tested forces behavior change. Clinicians randomized to receive a nudge are made aware of their high-risk patients and their performance compared to peers. Patients randomized to receive a nudge will have the option to complete an electronic questionnaire that may prime them for a SIC. Neither clinicians nor patients will be coerced or forced to engage in SICs; rather, the goal of nudges is to make standard evidence-based practices easier to conduct. Finally, as part of a previous quality improvement initiative, we interviewed 40 patients after SICs with their oncologist. We found no evidence of harm and found that serious illness conversations were considered by patients to be standard of care for patients with cancer.

## *4. Early Withdrawal of Subjects:*

We are requesting a waiver of informed consent, so the option to withdraw early from this study is not applicable.

***5. Vulnerable Populations:***

Children, pregnant women, fetuses, neonates, or prisoners are not being targeted in this research study.

***6. Populations vulnerable to undue influence or coercion:***

We will not be targeting participants who are likely to be vulnerable to undue influence or coercion.

**STUDY DESIGN:**

1. ***Study design:***

We will conduct a pragmatic four-arm randomized controlled trial to test the effectiveness of nudges to clinicians, nudges to patients, or nudges to both in promoting SIC documentation, compared to usual practice. We will independently randomize eligible clinicians and patients using a 2x2 factorial design. Clinicians will be randomized to receive a nudge consisting of targeted text messages identifying patients at high risk of predicted 6-month mortality based on a validated machine learning prognostic algorithm and performance feedback compared to peers (“clinician peer comparison”), versus usual practice. Patients will be randomized to receive a nudge consisting of a normalizing message prompting patients to complete an electronic questionnaire designed to prime patients towards having an SIC (“patient priming”), versus usual practice. This design will thus lead to four independent study groups: (1) nudge to both clinician and patient, (2) nudge to clinician only, (3) nudge to patient only, and (4) usual practice, as depicted below.

**Figure: 2x2 factorial design**

|  |  | **Patient** | |
| --- | --- | --- | --- |
|  |  | Nudge | No nudge |
| **Clinician** | Nudge | **Arm 1**: Nudge to clinician and patient (peer comparison & patient priming) | **Arm 2**: Nudge to clinician only  (clinician peer comparison) |
|  | No nudge | **Arm 3**: Nudge to patient only  (patient priming) | **Arm 4**: Usual practice  (active control) |

1. ***Study Duration:***

The study duration will be approximately 6 months per participant, defined by an initial index clinical encounter (marking the beginning of the nudge exposure period) and followed by a 6-month follow-up period over which outcomes will be ascertained. We will employ rapid cycle approaches (RCA) prior to clinical trial enrollment to optimize implementation strategies.

**METHODS:**

1. ***Study Instruments:***

Any study instruments will be submitted to the IRB as a modification prior to use.

## *Administration of Surveys and/or Process:*

Any surveys will be submitted to the IRB as a modification prior to use.

***3. Data Management:***

To minimize the risk of breach of data and confidentiality, we will use secure, encrypted servers to host the data and conduct the analysis. The Penn Medicine Academic Computing Services (PMACS) will be the hub for the hardware and database infrastructure that will support the project. The PMACS is a joint effort of the University of Pennsylvania's Abramson Cancer Center, the Cardiovascular Institute, the Department of Pathology, and the Leonard Davis Institute. The PMACS provides a secure computing environment for a large volume of highly sensitive data, including clinical, genetic, socioeconomic, and financial information. PMACS requires all users of data or applications on PMACS servers to complete a PMACS-hosted cybersecurity awareness course annually, which stresses federal data security policies under data use agreements with the university. The curriculum includes Health Insurance Portability and Accountability Act (HIPAA) training and covers secure data transfer, passwords, computer security habits and knowledge of what constitutes misuse or inappropriate use of the server. We will implement multiple, redundant protective measures to guarantee the privacy and security of the participant data. All investigators and research staff with direct access to the identifiable data will be required to undergo annual responsible conduct of research, cybersecurity, and HIPAA certification in accordance with University of Pennsylvania regulations. Data will be stored, managed, and analyzed on a secure, encrypted server behind the University of Pennsylvania Health System (UPHS) firewall. All study personnel that will use this data are listed on the IRB application and have completed training in HIPAA standards and the CITI human subjects research. Data access will be password protected. Whenever possible, data will be deidentified for analysis.

## *4. Subject Follow-up:*

## Subjects will be followed for approximately 6-months after enrollment.

## STUDY PROCEDURES:

***1. Detailed Description:***

Eligible clinicians and patients will be identified using the criteria listed above, and will be independently randomized to study arms using a 2x2 factorial design. Eligible clinicians will be randomized *at the start of the study* and will receive nudges identifying their high-risk patients with or without peer comparisons for the duration of the study period. Eligible patients will be randomized *in advance of an index outpatient clinical encounter*, and will accrue to the study accordingly. Patients will be exposed to implementation strategies based on their assigned study arm, which will depend on their own randomization and that of their clinician (determined at the time of the index outpatient clinical encounter). Physicians will be mapped to advanced practitioners (i.e. physician assistants, nurse practitioners) with whom they work, and vice versa, and this cluster will serve as the clinician unit of analysis and thus will be cluster randomized.

*Nudge to clinician only*. The clinician nudge will consist of targeted text messages identifying patients at high risk of predicted 6-month mortality based on a validated machine learning prognostic algorithm,^33,34^ as well as SIC documentation performance feedback graphically compared to peers (“clinician peer comparison”). The clinician nudge will be delivered weekly via email with text messages sent on the morning(s) before clinic sessions. See **Appendix 1. Sample Provider Nudge** for the content of provider nudge email and text.

*Nudge to patient only*. The patient nudge will consist of a normalizing message prompting patients to complete an electronic questionnaire designed to prime patients towards having a SIC (“patient priming”). The nudge will be delivered via text message and email in advance of the index clinical encounter, and will be re-sent a maximum of two times at monthly intervals only for those patients who neither fill out the priming questionnaire nor have a documented SIC during follow-up. Patient-reported data from the priming questionnaire will be shared with clinical teams in real-time. See **Appendix 2. Sample Patient Nudge** for the content of patient nudge texts, email, and electronic questionnaire.

*Nudges to both clinician and patient*. Both strategies described above will be used.

*Usual practice*. Usual practice will consist of targeted text messages identifying patients at high risk of mortality based on a validated machine learning prognostic algorithm, as well as clinician-directed performance feedback (WITHOUT peer comparison), thereby serving as an active control arm.

We will use RCA to optimize implementation strategies to ensure face validity and maximum effect. We will focus on optimizing content, messaging, and design. RCA procedures will involve design meetings with study team, discussions with administrators and clinicians who are members of our Implementation Lab, as well as piloting of nudges with clinicians and patients to elicit feedback.

***2. Data Collection:***

The electronic medical record and other Penn Medicine secondary databases will be used to collect information on practices, clinicians, and patients. These systems collect this information as part of routine clinical care. We will also collect data from the U.S. Census via publicly available datasets.

***Aim 1***

The primary outcome is SIC documentation among high-risk patients. This will be measured at the patient level as a binary outcome, ascertained over the 6-month period following the index outpatient clinical encounter. The electronic medical record has an Advanced Care Planning (ACP) section in which ACP notes are documented. Any note documented in this section will be classified as an ACP, and only the subset of notes that include a preformatted SIC template will be classified as a SIC.

The secondary outcomes will each be measured at the patient level as a binary outcome, ascertained over the 6-month period following the index outpatient clinical encounter:

- SIC documentation, among all patients
- Palliative care referral, among high-risk patients
- Aggressive end-of-life care (any of the following three criteria: chemotherapy within 14 days before death, hospitalization within 30 days before death, or admission to hospice 3 days or less before death), among high-risk patients who die

***Aim 2***

Aim 2 measures, collected through the electronic medical record and using publically available U.S. Census data, will include:

- *Patient-level data*: age, sex, race/ethnicity, cancer type, cancer stage, health insurance (e.g., Medicare, Medicaid, commercial);
- *Clinician-level data*: years in practice and patient panel size;
- *Practice-level data*: setting (community vs. hospital-based), urban vs. non-urban location, and health insurance mix;
- *Ecologic-level data* linked at the patient- and practice-level, including median income and educational attainment.

***3. Genetic Testing:***

Not applicable

***4. Use of Deception:***

Not applicable

***5. Statistical Analysis:***

***Aim 1***

The primary outcome will be modeled in a time-to-event analysis using a Cox proportional hazards model, with cluster-correlated robust standard errors to account for clustering by clinical site. SIC documentation, as defined above, will serve as the primary event outcome. Days between the index clinical encounter (at which the nudge exposure period begins) and SIC documentation will serve as the time variable. The design is a 2x2 factorial, which breaks down into 4 study arms (defined by clinician cluster and patient randomization at the index clinical encounter): nudge to clinician only, nudge to patient only, nudge to both, or usual practice. Cox regression will estimate hazard ratios for the main effects (Aim 1) and a ratio of hazard ratios for the interaction term (Aim 2). Significance will be determined using the z-score corresponding to each of the estimated effects, using a two sided type 1 error of 5%. All enrolled patients will be included in the intention-to-treat analysis, and subjects will be censored at the time of last follow-up or death, should they not have been observed to have a documented SIC during the follow-up period. Covariates will be assessed across study arms and included in the Cox model if unbalanced across arms.

We will use our fitted Cox model to generate predicted probabilities of SIC documentation within 6 months, via marginal standardization, as well as median time to documented SIC across arms. The functional form of the model will be checked using cumulative martingale residuals, and the proportional hazards assumption will be checked using scaled Schoenfeld residuals.

The secondary outcomes will be similarly modeled. All hypothesis tests will use a two-sided alpha of 0.05 as the threshold for statistical significance.

***Aim 2***

We will evaluate for heterogeneity of implementation effects on SIC documentation by including an interaction term between study arm and, separately, patient factors, such as race/ethnicity, income, and geographic location; clinician factors; and inner setting factors. Evidence for effect modification will be judged based on the z-score corresponding to the ratio of hazard ratios (interaction term) using again a two-sided alpha of 0.05 as the threshold for statistical significance.

***Power and Sample Size***

We are seeking to detect the main effects of nudges and their interaction with at least 80% power using a two-sided alpha of 0.05. We anticipate enrolling 66 physician-APP clusters who will together care for an estimated 5,500 high-risk patients over the study period, yielding approximately 83 high-risk patients per cluster on average. We have generated power calculations using various patient enrollment and cluster correlation assumptions as below. We calculated power by simulation, generating exponential time to event under a variety of assumptions. Patients are clustered within clinician units. Higher correlation within cluster will generally help with power for within-cluster comparisons, but degrade power for between-cluster comparisons. Within cluster correlation was imposed by drawing correlated Normally-distributed random numbers, transformed to exponential using copula methods.

| Patients per Cluster | Within Cluster Correlation | Detectable True HR of Effect (Power) | | |
| --- | --- | --- | --- | --- |
|  |  | Provider nudge | Patient nudge | Interaction |
| 70 | 0.1 | 1.6 (86%) | 1.25 (84%) | 1.6 (80%) |
| 70 | 0.3 | 2.0 (85%) | 1.3 (90%) | 1.8 (91%) |
| 90 | 0.1 | 1.6 (91%) | 1.25 (86%) | 1.6 (85%) |
| 90 | 0.3 | 2.0 (86%) | 1.25 (84%) | 1.6 (83%) |

Assuming conservatively that we enroll 70 patients per cluster with a cluster correlation of 0.1, we will have >80% power to detect a true hazard ratio of effect of HR 1.6 for the provider nudge, HR 1.25 for the patient nudge, and HRR 1.6 for the interaction. Assuming 70 patients per cluster and a cluster correlation of 0.3, we will have >80% power to detect a true hazard ratio of effect of HR 2.0 for the provider nudge, HR 1.3 for the patient nudge, and HRR 1.8 for the interaction. These estimates improve slightly if we assume enrollment of 90 patients per cluster, allowing us to detect similar effect sizes with more power, as shown in the Table above.

**RISK/BENEFIT ASSESSMENT:**

***1. Risks:***

There are minimal risks to participants in this trial. There is a risk of breach of data and confidentiality, however we described the precautions in place to securely manage this data in the Data Management section of this protocol. As noted above, substantial data demonstrates that ACPs improve patient goal-concordant care without any identified harms (despite concerns that ACPs may increase psychosocial distress, the opposite has been found), so the negative impact on patients is minimal.

The provider data that will be shared with providers is already shared in one form (in the case of SIC rates), so the trial does not expose providers to additional risk.

***2. Benefits:***

As described in the literature, patients may have improved quality of life and better goal-concordant care when exposed to ACPs, especially earlier in their disease course. An intervention that prompts providers to have an SIC with patients on active treatment for cancer may increase the likelihood that these conversations occur and that they occur earlier in the disease course. However, it is possible that patients will receive no benefit from this study.

***3. Subject Privacy:***

## Privacy will be given utmost consideration and is highly valued in the proposed research. No research activities involve any direct interaction with subjects that would pose risk to their privacy.

## *4. Subject Confidentiality:*

## Confidentiality refers to the subject’s understanding of, and agreement to, the ways in which identifiable information will be stored and shared.

**How will confidentiality of data be maintained?**

Paper-based records will be kept in a secure location and only be accessible to personnel involved in the study.

Computer-based files will only be made available to personnel involved in the study through the use of access privileges and passwords.

Prior to access to any study-related information, personnel will be required to sign statements agreeing to protect the security and confidentiality of identifiable information.

Whenever feasible, identifiers will be removed from study-related information.

A Certificate of Confidentiality will be obtained, because the research could place the subject at risk of criminal or civil liability or cause damage to the subject’s financial standing, employability, or liability.

A waiver of documentation of consent is being requested, because the only link between the subject and the study would be the consent document and the primary risk is a breach of confidentiality. (This is not an option for FDA-regulated research.)

Precautions are in place to ensure the data is secure by using passwords and encryption, because the research involves web-based surveys.

Audio and/or video recordings will be transcribed and then destroyed to eliminate audible identification of subjects.

Other (specify):

To protect participant confidentiality, only the research team outlined in HSERA will have access to review identified research records. Confidentiality will be protected to the fullest extent allowable under the law. See the Data Management section for more details.

If any data needs to be transmitted, it will be done through a Penn-approved secure encrypted file transfer solution as is described Penn IRB’s Guidance on Electronic Data Protection Requirements for Research Involving the Use of PHI. Records will not be released without the participant’s consent unless required by law (e.g., imminent risk of harm to self suspected) or court order. When results of the research are presented at scientific meetings or published, no identifying information will be included.

All identifiable data, including the master list linking identifiers to the ID number and recordings, will be destroyed in 2028, seven years after the award period ends.

***5. Protected Health Information***

- Name
- Address
- Date of Birth
- Phone number(s)
- Electronic mail address

***6. Compensation:***

Participants will not be compensated for participating in this study.

***7. Data and Safety Monitoring:***

The nature of the project poses minimal risk to participant safety and privacy. Yet, we will constitute a formal Data Safety Monitoring Board. The specific aspects of the DSMB for this study are as follows:

1. The DSMB will consist of 4 members: 1) Erin Aakhus, MD, Assistant Professor of Clinical Medicine, Perelman School of Medicine, University of Pennsylvania, Associate Director of the Hematology Oncology Fellowship Program 2) Kate Courtright, MD, MSHP, Assistant Professor of Clinical Medicine at the Perelman School of Medicine of the University of Pennsylvania 3) Kit Delgado, MD, MS, Assistant Professor of Emergency Medicine at the Perelman School of Medicine of the University of Pennsylvania, Associate Director of Center for Health Incentives and Behavioral Economics 4) Meghan Lane-Fall, MD, MSHP, David E. Longnecker Associate Professor of Anesthesiology and Critical Care & Associate Professor of Epidemiology at the Perelman School of Medicine of the University of Pennsylvania, Associate Director of the Center for Health Incentives and Behavioral Economics
2. The DSMB will perform several duties. First, they will review and approve research protocols and plans for data and safety monitoring prior to any study commencement. Second, they will evaluate the progress of any eligible trial. This will include assessment of data quality, participant recruitment, accrual and retention, participant risk versus benefit, and study outcomes. This assessment will be performed at meetings every six months during eligible trials and, more frequently, if decided by the DSMB. Third, they will make recommendations to ensure that all of the issues above are appropriately addressed. The corresponding project teams will be responsible for responding to all recommendations of the DSMB and submitting DSMB reports to the University of Pennsylvania IRB.

*Data Safety and Monitoring Plan.* Oversight and evaluation will be accomplished using standard University procedures for safety monitoring. The specific elements of our oversight plan are as above: 1) all project staff will complete certification in the protection of research participants; 2) the principal investigator will supply the IRB with annual progress reports, or more frequently as determined by the IRB, which may in turn suspend, terminate or restrict the study as appropriate; and 3) any serious adverse events will be reviewed in real time by the PI and reported to the IRB as required. Individual-level data for participants will be kept confidential and will only be stored on highly secure servers available for patient-level data. Only authorized project personnel will have access to the data and the data will only be stored on servers and not stand-alone PCs or laptops. All data will be reported at units of aggregation which make impossible the identification of individual patients or clinicians.

The data and safety monitoring plan will have 3 parts. First, the study MPIs, biostatistician, and Director of the Data Management Unit will develop and implement methods of verifying entered data and of quality control. Second, the MPIs will be directly responsible for identifying and reporting all adverse events, protocol deviations/violations and unanticipated events to the IRB and funding agency promptly, as appropriate. The PIs will also report all adverse events, accrual rates, retention rates, and all other logistical issues to the DSMB (described above) at least biannually (and more frequently if there are serious adverse events). Third, there will be a DSMB responsible for monitoring the trial.

A written research protocol will undergo formal institutional scientific and institutional review board (IRB) review at the University of Pennsylvania (Penn) to ensure protection of the rights and welfare of human research subjects. Specifically, the MPIs and the IRB will be responsible for ensuring risks to human subjects are minimized, risks are reasonable, subject selection is equitable, the research team has access to adequate resources to conduct the study, the informed consent process (or waiver) meets regulatory and ethical requirements, adequate provision is made to protect human subjects by monitoring the data collected and there are adequate provisions to protect subject privacy per HIPAA regulations and confidentiality of data.

All senior/key personnel and research staff who will be involved in the design and conduct of the study must receive education in human research subject protection from a training program that is approved by a properly constituted independent Ethics Committee or Institutional Review Board. The MPIs will be responsible for ensuring project faculty and staff have the equipment and training required to protect privacy and confidentiality and will monitor and document that these individuals are properly certified. If new senior/key personnel and staff become involved in the research, documentation that they have received the required education will be included in the annual progress reports. The UPENN Office of Regulatory Affairs currently requires HIPAA training upon designation as research investigator/staff and recertification in human research subjects protection every three years.

The Penn IRB will serve as the IRB of record for any external ethics review boards or IRBs applicable to researchers from other institutions who may have access to human research subjects identified data.

***8. Investigator’s Risk/Benefit Assessment:***

This study presents minimal risk that is balanced by the potential benefits of the research to society.

**INFORMED CONSENT:**

***1. Consent Process:***

Aims 1 and 2. Since this is a pragmatic trial focused on improving implementation of higher-value evidence-based practices with minimal risk to patients, we are requesting a waiver of informed consent from clinicians and patients. We have received this in the past for these types of trials. We will identify clinicians at the practice sites and their patients using the EMR.

**2. Waiver of Informed Consent:**

We are requesting a waiver of informed consent and HIPAA authorization from clinicians and patients (see attached request for waiver of HIPAA authorization). A waiver of informed consent is requested for the following reasons. First, it is not feasible to consent every patient and clinician randomized to nudges. Second, if members of the control group were consented, they would know they were being studied and this could change their behavior. This could potentially disrupt the design of the study and make interpretation of the findings challenging. Third, clinicians are not forced to have serious illness conversations with their patients, and patients are not being forced to engage in the pre-work or the conversations with their providers. In all arms, clinicians can still engage or refrain from the conversation according to their best clinical judgment, and patients can refuse to participate in the conversation, even when engaged by their clinician. Finally, as part of a previous quality improvement initiative, we interviewed 40 patients after a serious illness conversation with their oncologist. We found no evidence of harm and found that serious illness conversations were considered standard of care for patients with cancer. This has been reiterated in the previous study, during which no clinicians reported harm as a result of the intervention.

**RESOURCES NECESSARY FOR HUMAN RESEARCH PROTECTION:**

Adequate facilities are available at the ACC. The members of the research team are outlined in HSERA and include appropriate personnel to successfully implement this project. The entire team will be overseen by the PI. All personnel will complete required training before being granted access to any identifying information. This includes training on confidentiality through the Collaborative IRB Training Initiative (CITI) course. All personnel will be trained in the procedures for reporting unintentional breaches in confidentiality to the PI. All personnel will be aware that violations of participants’ confidentiality, either unintentional or deliberate, may result in termination of hire. The PI will conduct training with all research personnel regarding data, limits of confidentiality, maintaining confidentiality and proper study procedures.

The following research staff will be directly involved with the implementation and execution of the current study:

| **Name** | **Study Role** |
| --- | --- |
| Ravi Parikh, MD, MPP, FACP | Principal Investigator |
| Samuel U. Takvorian, MD, MS | Principal Investigator |
| Robert A. Schnoll, PhD | Collaborator |
| Rinad Beidas, PhD | Collaborator |
| Justin Bekelman, MD | Collaborator |
| Peter Gabriel, MD, MSE | Informatics |
| Alicia Clifton, MDP | Project Manager |
| Tasnim Salam, MBE, MPH | Clinical Research Coordinator |
| Sue Ware, BS | Database Manager |
| Paul Wileyto, PhD | Data Analyst |

**REFERENCES**

1. Earle CC, Landrum MB, Souza JM, Neville BA, Weeks JC, Ayanian JZ. Aggressiveness of cancer care near the end of life: is it a quality-of-care issue? *J Clin Oncol Off J Am Soc Clin Oncol*. 2008;26(23):3860-3866. doi:10.1200/JCO.2007.15.8253

2. Emanuel EJ, Young-Xu Y, Levinsky NG, Gazelle G, Saynina O, Ash AS. Chemotherapy use among Medicare beneficiaries at the end of life. *Ann Intern Med*. 2003;138(8):639-643. doi:10.7326/0003-4819-138-8-200304150-00011

3. Wright AA, Keating NL, Ayanian JZ, et al. Family Perspectives on Aggressive Cancer Care Near the End of Life. *JAMA*. 2016;315(3):284-292. doi:10.1001/jama.2015.18604

4. Wright AA, Keating NL, Balboni TA, Matulonis UA, Block SD, Prigerson HG. Place of death: correlations with quality of life of patients with cancer and predictors of bereaved caregivers’ mental health. *J Clin Oncol Off J Am Soc Clin Oncol*. 2010;28(29):4457-4464. doi:10.1200/JCO.2009.26.3863

5. Wen F-H, Chen J-S, Su P-J, et al. Terminally Ill Cancer Patients’ Concordance Between Preferred Life-Sustaining Treatment States in Their Last Six Months of Life and Received Life-Sustaining Treatment States in Their Last Month: An Observational Study. *J Pain Symptom Manage*. 2018;56(4):509-518.e3. doi:10.1016/j.jpainsymman.2018.07.003

6. Earle CC, Neville BA, Landrum MB, Ayanian JZ, Block SD, Weeks JC. Trends in the aggressiveness of cancer care near the end of life. *J Clin Oncol Off J Am Soc Clin Oncol*. 2004;22(2):315-321. doi:10.1200/JCO.2004.08.136

7. Wright AA, Zhang B, Ray A, et al. Associations between end-of-life discussions, patient mental health, medical care near death, and caregiver bereavement adjustment. *JAMA*. 2008;300(14):1665-1673. doi:10.1001/jama.300.14.1665

8. Mack JW, Weeks JC, Wright AA, Block SD, Prigerson HG. End-of-life discussions, goal attainment, and distress at the end of life: predictors and outcomes of receipt of care consistent with preferences. *J Clin Oncol Off J Am Soc Clin Oncol*. 2010;28(7):1203-1208. doi:10.1200/JCO.2009.25.4672

9. Detering KM, Hancock AD, Reade MC, Silvester W. The impact of advance care planning on end of life care in elderly patients: randomised controlled trial. *BMJ*. 2010;340:c1345. doi:10.1136/bmj.c1345

10. Brinkman-Stoppelenburg A, Rietjens JAC, van der Heide A. The effects of advance care planning on end-of-life care: a systematic review. *Palliat Med*. 2014;28(8):1000-1025. doi:10.1177/0269216314526272

11. Bernacki R, Paladino J, Neville BA, et al. Effect of the Serious Illness Care Program in Outpatient Oncology: A Cluster Randomized Clinical Trial. *JAMA Intern Med*. 2019;179(6):751-759. doi:10.1001/jamainternmed.2019.0077

12. Paladino J, Bernacki R, Neville BA, et al. Evaluating an Intervention to Improve Communication Between Oncology Clinicians and Patients With Life-Limiting Cancer: A Cluster Randomized Clinical Trial of the Serious Illness Care Program. *JAMA Oncol*. 2019;5(6):801-809. doi:10.1001/jamaoncol.2019.0292

13. Bickel KE, McNiff K, Buss MK, et al. Defining High-Quality Palliative Care in Oncology Practice: An American Society of Clinical Oncology/American Academy of Hospice and Palliative Medicine Guidance Statement. *J Oncol Pract*. 2016;12(9):e828-838. doi:10.1200/JOP.2016.010686

14. Bernacki RE, Block SD, American College of Physicians High Value Care Task Force. Communication about serious illness care goals: a review and synthesis of best practices. *JAMA Intern Med*. 2014;174(12):1994-2003. doi:10.1001/jamainternmed.2014.5271

15. *Committee on Approaching Death: Addressing Key End-Of-Life Issues*. National Academies Press (US); 2015. Accessed September 4, 2020. https://www.ncbi.nlm.nih.gov/books/NBK285678/

16. Schubart JR, Levi BH, Bain MM, Farace E, Green MJ. Advance Care Planning Among Patients With Advanced Cancer. *J Oncol Pract*. 2019;15(1):e65-e73. doi:10.1200/JOP.18.00044

17. Rao JK, Anderson LA, Lin F-C, Laux JP. Completion of Advance Directives Among U.S. Consumers. *Am J Prev Med*. 2014;46(1):65-70. doi:10.1016/j.amepre.2013.09.008

18. Degenholtz HB, Arnold RA, Meisel A, Lave JR. Persistence of Racial Disparities in Advance Care Plan Documents Among Nursing Home Residents. *J Am Geriatr Soc*. 2002;50(2):378-381. doi:10.1046/j.1532-5415.2002.50073.x

19. Kulkarni SP, Karliner LS, Auerbach AD, Pérez-Stable EJ. Physician Use of Advance Care Planning Discussions in a Diverse Hospitalized Population. *J Immigr Minor Health*. 2011;13(3):620-624. doi:10.1007/s10903-010-9361-5

20. Smith AK, McCarthy EP, Paulk E, et al. Racial and Ethnic Differences in Advance Care Planning Among Patients With Cancer: Impact of Terminal Illness Acknowledgment, Religiousness, and Treatment Preferences. *J Clin Oncol*. 2008;26(25):4131-4137. doi:10.1200/JCO.2007.14.8452

21. LoPresti MA, Dement F, Gold HT. End-of-Life Care for People With Cancer From Ethnic Minority Groups: A Systematic Review. *Am J Hosp Palliat Med*. 2016;33(3):291-305. doi:10.1177/1049909114565658

22. Takvorian SU, Ladage VP, Wileyto EP, et al. Association of Behavioral Nudges With High-Value Evidence-Based Prescribing in Oncology. *JAMA Oncol*. Published online April 30, 2020. doi:10.1001/jamaoncol.2020.0746

23. Meeker D, Linder JA, Fox CR, et al. Effect of Behavioral Interventions on Inappropriate Antibiotic Prescribing Among Primary Care Practices: A Randomized Clinical Trial. *JAMA*. 2016;315(6):562-570. doi:10.1001/jama.2016.0275

24. Patel MS, Day SC, Halpern SD, et al. Generic Medication Prescription Rates After Health System-Wide Redesign of Default Options Within the Electronic Health Record. *JAMA Intern Med*. 2016;176(6):847-848. doi:10.1001/jamainternmed.2016.1691

25. Patel MS, Volpp KG, Asch DA. Nudge Units to Improve the Delivery of Health Care. *N Engl J Med*. 2018;378(3):214-216. doi:10.1056/NEJMp1712984

26. Manz C, Parikh RB, Evans CN, et al. Effect of integrating machine learning mortality estimates with behavioral nudges to increase serious illness conversions among patients with cancer: A stepped-wedge cluster randomized trial. *J Clin Oncol*. 2020;38(15_suppl):12002-12002. doi:10.1200/JCO.2020.38.15_suppl.12002

27. Chandar M, Brockstein B, Zunamon A, et al. Perspectives of Health-Care Providers Toward Advance Care Planning in Patients With Advanced Cancer and Congestive Heart Failure. *Am J Hosp Palliat Care*. 2017;34(5):423-429. doi:10.1177/1049909116636614

28. Christakis NA, Lamont EB. Extent and determinants of error in doctors’ prognoses in terminally ill patients: prospective cohort study. *BMJ*. 2000;320(7233):469-472. doi:10.1136/bmj.320.7233.469

29. Sborov K, Giaretta S, Koong A, et al. Impact of Accuracy of Survival Predictions on Quality of End-of-Life Care Among Patients With Metastatic Cancer Who Receive Radiation Therapy. *J Oncol Pract*. 2019;15(3):e262-e270. doi:10.1200/JOP.18.00516

30. Kiefe CI, Allison JJ, Williams OD, Person SD, Weaver MT, Weissman NW. Improving quality improvement using achievable benchmarks for physician feedback: a randomized controlled trial. *JAMA*. 2001;285(22):2871-2879. doi:10.1001/jama.285.22.2871

31. Linder JA, Meeker D, Fox CR, et al. Effects of Behavioral Interventions on Inappropriate Antibiotic Prescribing in Primary Care 12 Months After Stopping Interventions. *JAMA*. 2017;318(14):1391-1392. doi:10.1001/jama.2017.11152

32. Cohn A, Maréchal MA. *Priming in Economics*. Social Science Research Network; 2016. doi:10.2139/ssrn.2775289

33. Parikh RB, Manz C, Chivers C, et al. Machine Learning Approaches to Predict 6-Month Mortality Among Patients With Cancer. *JAMA Netw Open*. 2019;2(10):e1915997. doi:10.1001/jamanetworkopen.2019.15997

34. Manz CR, Chen J, Liu M, et al. Validation of a Machine Learning Algorithm to Predict 180-Day Mortality for Outpatients With Cancer. *JAMA Oncol*. 2020;6(11):1723. doi:10.1001/jamaoncol.2020.4331

**APPENDICES**

1. ***Sample Provider Nudge***

*Sample timeline of provider nudge deployment*

Week 1

- Thursday:
  - Eligible patients identified via machine learning algorithm
  - Provider-level randomization; Provider email delivered

Week 2

- Monday-Friday: Provider text delivered morning of index repeat patient visit (RPV)

*Sample provider email content*

Arms 3 (Patient Nudge Only) & 4 (Active Control):

Dear XXX:

The ACC is working to help oncologists have earlier Serious Illness Conversations with patients.

In the past four weeks, you have documented 3 conversations.

We have identified patients scheduled to see you next week who may benefit from a Serious Illness Conversation. Click here to view your list (you must be connected to the UPHS network).

Sincerely,

[Abramson Cancer Center Leadership]

Arms 1 (Both Patient & Clinician Nudge) & 2 (Clinician Nudge Only)


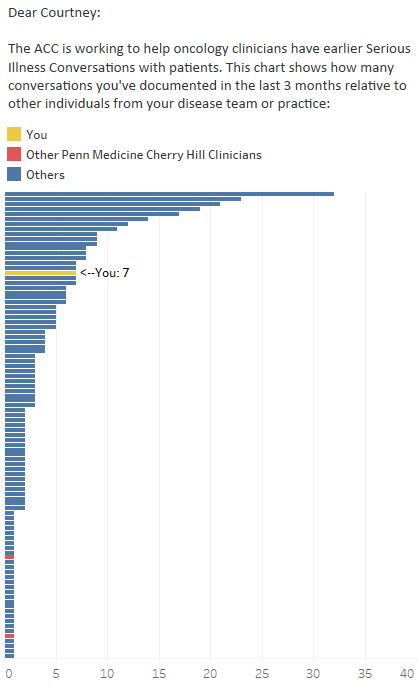


We have identified patients scheduled to see you next week who may benefit from a Serious Illness Conversation. Click here to view your list (you must be connected to the UPHS network).

Sincerely,

[Abramson Cancer Center Leadership]

*Sample provider text content (All Arms)*

The following patients may be appropriate for a Serious Illness Conversation:

A.B. 10 AM

B.C. 11 AM

1. ***Sample Patient Nudge***

*Sample timeline of patient nudge deployment*

Week 1

- Thursday: Eligible patients identified via machine learning algorithm

Friday: Patient-level randomization; Patient nudges delivered

Week 2

- Monday-Friday: Index repeat patient visit (RPV)

*Sample texts*


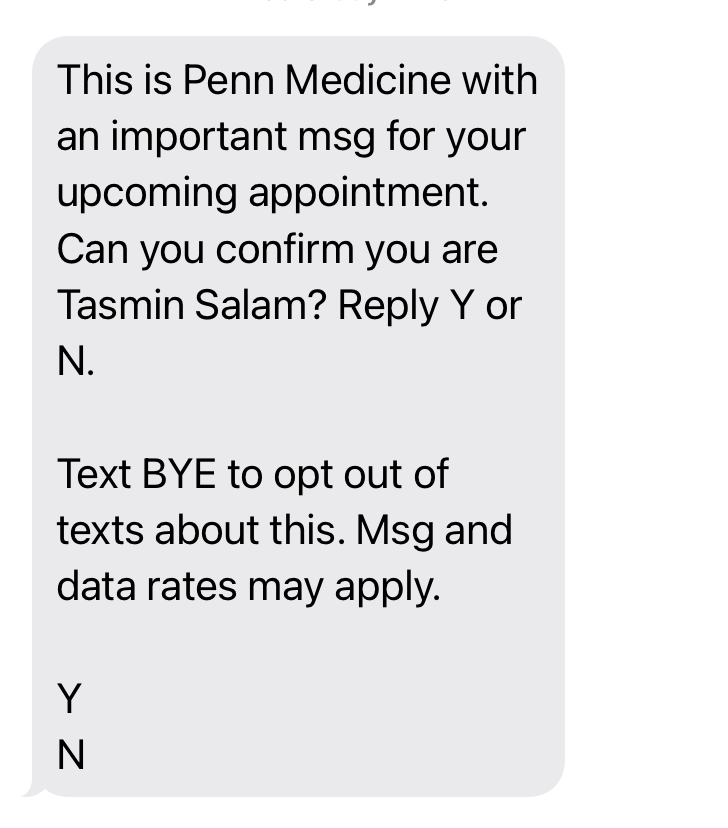

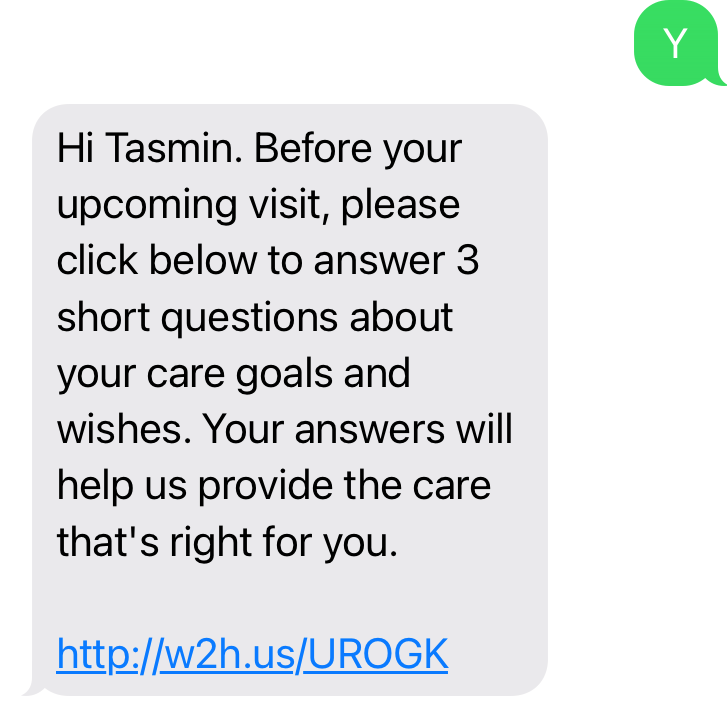


*Sample email*


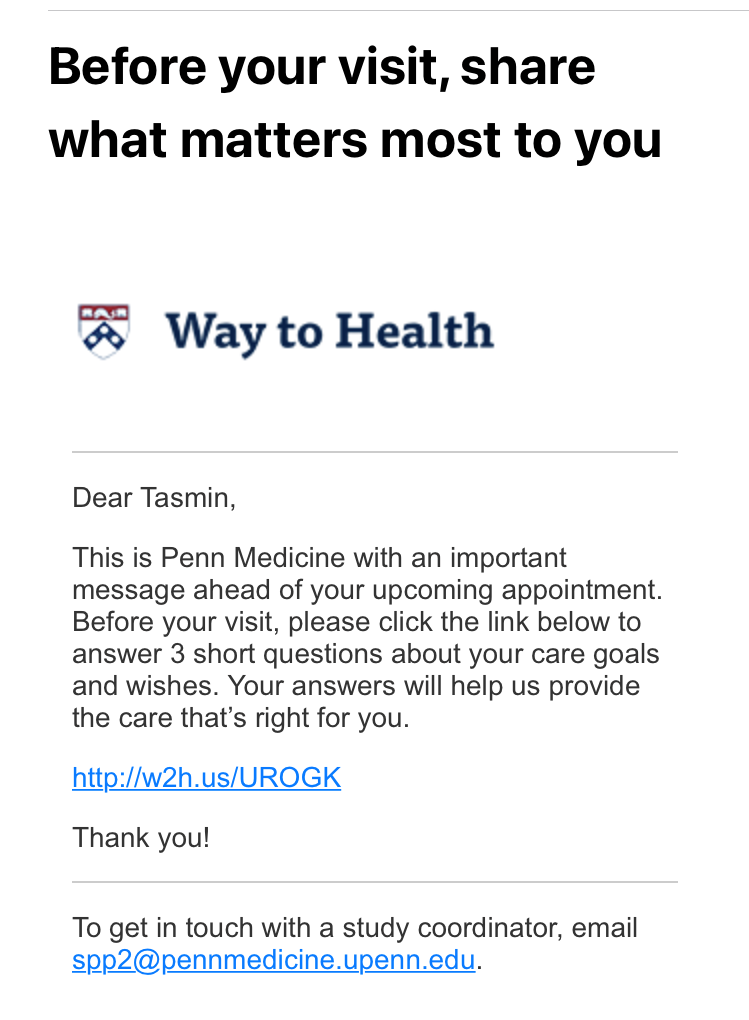


*Sample electronic patient questionnaire*


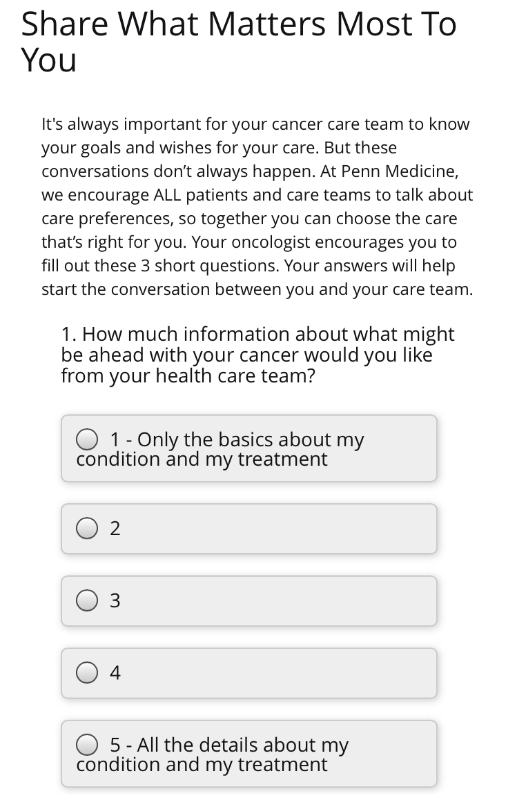


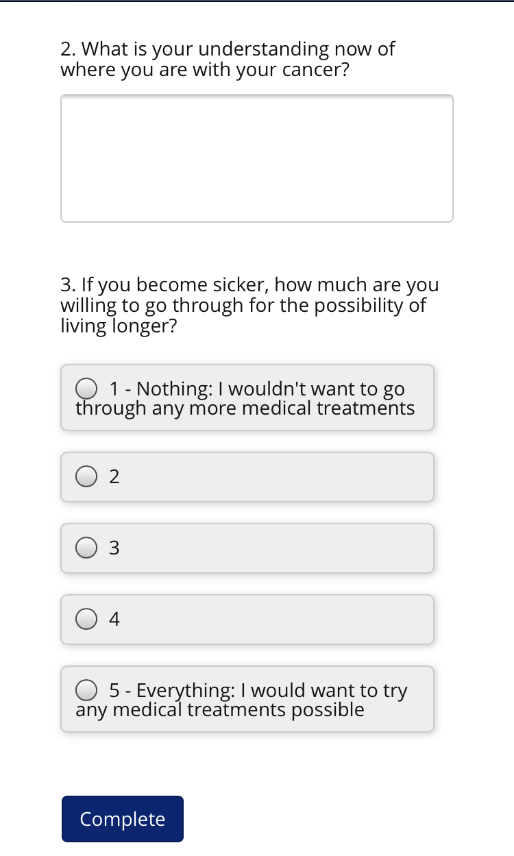

Supplement: Supplementary file 2 — Additional file 2. Full Protocol-SIC Protocol. Title: Social and Behavioral Sciences Human Research Protocol. Description: IRB-approved study protocol [file 13012_2021_1156_MOESM2_ESM.docx]
