# Supplementary material for: Behavioral economic implementation strategies to improve serious illness communication between clinicians and high-risk patients with cancer: protocol for a cluster randomized pragmatic trial
Source: Implement Sci. 2021 Sep 25;16:90. doi: 10.1186/s13012-021-01156-6 (PMC8466719; doi:10.1186/s13012-021-01156-6)
Supplement: Supplementary file 4 — Additional file 4. IRB Approval Letter-SIC Protocol. Title: IRB Amendment: Notice of Approval. Description: IRB approval letter [file 13012_2021_1156_MOESM4_ESM.pdf]

Institutional Review Board
3600 Civic Center Blvd., 9th Floor
Philadelphia, PA 19104
Phone: 215-573-2540
(Federalwide Assurance # 00004028)

DATE:	28-Jul-2021
TO:	Samuel Takvorian
CC:	Lieberman, Adina E<br>Salam, Tasnim<br>Clifton, Alicia
RE:	
IRB PROTOCOL#:	844816
PROTOCOL TITLE:	Effect of behavioral nudges to clinicians, patients, or both on Serious Illness Conversation documentation for patients with cancer
SPONSOR:	National Cancer Institute/NIH/DHHS
REVIEW BOARD:	IRB #8

IRB AMENDMENT: NOTICE OF APPROVAL

Dear Dr. Takvorian,

The documents noted below, for the above-referenced protocol, were reviewed by the Institutional Review Board using the expedited procedure set forth in 45 CFR 46.110 and approved on 27-Jul-2021.

Consistent with the regulations set forth in 45 CFR 46.109(f), continuing review of this research is not required. IRB approval of this protocol will not expire and continuing review applications should not be submitted. However, you are still required to submit modifications and reportable events to the IRB for review.

The documents included with the application noted below are approved:
-HSERA modification submission (confirmation # deddbeee) submitted 7/26/2021
ONGOING REQUIREMENTS:

- You must obtain IRB review and approval under 45 CFR 46 if you make any changes to the protocol, consent form, or any other study documents subject to IRB review requirements. Implementation of any changes cannot occur until IRB approval has been given.
- Reportable event, such as serious adverse events, deviations, potential unanticipated problems, and reports of non-compliance must be reported to the IRB in accordance with Penn IRB SOP RR 404.
- When enrolling subjects at a site covered by the University of Pennsylvania's IRB, a copy of the IRB approved informed consent form

with the IRB approved from/to stamp must be used unless a waiver of written documentation of consent has been granted.

COMMITTEE APPROVALS: You are responsible for assuring and maintaining other relevant committee approvals. This human subjects research protocol should not commence until all relevant committee approvals have been obtained.

If your study is funded by an external agency, please retain this letter as documentation of the IRB's determination regarding your proposal.

If you have any questions about the information in this letter, please contact the IRB administrative staff. A full listing of staff members and contact information can be found on our website: http://www.irb.upenn.edu

***This letter constitutes official University of Pennsylvania IRB correspondence. ***
